# Brain networks underlying vulnerability and resilience to drug addiction

**DOI:** 10.1073/pnas.2002509117

**Published:** 2020-06-15

**Authors:** Karen D. Ersche, Chun Meng, Hisham Ziauddeen, Jan Stochl, Guy B. Williams, Edward T. Bullmore, Trevor W. Robbins

**Affiliations:** ^a^Department of Psychiatry, University of Cambridge, Cambridge CB2 0SZ, United Kingdom;; ^b^Behavioural and Clinical Neuroscience Institute, Department of Psychology, University of Cambridge, Cambridge CB2 3EB, United Kingdom;; ^c^Cambridgeshire and Peterborough National Health Service Foundation Trust, Cambridge CB21 5EF, United Kingdom;; ^d^Department of Kinanthropology and Humanities, Charles University, 16252 Prague, Czech Republic;; ^e^Department of Clinical Neurosciences, University of Cambridge, Cambridge CB2 3EB, United Kingdom;; ^f^Department of Psychology, University of Cambridge, Cambridge CB2 3EB, United Kingdom

**Keywords:** functional connectivity, cocaine, vulnerability, resilience, fMRI

## Abstract

Regular drug use can lead to addiction, but not everyone who takes drugs makes this transition. How exactly drugs of abuse interact with individual vulnerability is not fully understood, nor is it clear how individuals defy the risks associated with drugs or addiction vulnerability. We used resting-state functional MRI (fMRI) in 162 participants to characterize risk- and resilience-related changes in corticostriatal functional circuits in individuals exposed to stimulant drugs both with and without clinically diagnosed drug addiction, siblings of addicted individuals, and control volunteers. The likelihood of developing addiction, whether due to familial vulnerability or drug use, was associated with significant hypoconnectivity in orbitofrontal and ventromedial prefrontal cortical-striatal circuits—pathways critically implicated in goal-directed decision-making. By contrast, resilience against a diagnosis of substance use disorder was associated with hyperconnectivity in two networks involving 1) the lateral prefrontal cortex and medial caudate nucleus and 2) the supplementary motor area, superior medial frontal cortex, and putamen—brain circuits respectively implicated in top-down inhibitory control and the regulation of habits. These findings point toward a predisposing vulnerability in the causation of addiction, related to impaired goal-directed actions, as well as countervailing resilience systems implicated in behavioral regulation, and may inform novel strategies for therapeutic and preventative interventions.

## Significance

Resting-state functional connectivity provides novel insight into variations in neural networks associated with addiction to stimulant drugs in individuals with and without a family history of addiction, and both with and without personal drug use. An increased risk for addiction, either because of drug use or genetic/psychosocial vulnerability, is associated with hypoconnectivity in frontostriatal networks, which may weaken goal-directed decision-making. Resilience against addiction development, by contrast, is characterized by hyperconnectivity in two corticostriatal pathways, possibly reflecting compensatory responses in networks associated with regulatory control over habitual behaviors. It is thus conceivable that defying the risk of developing stimulant drug addiction requires increased efforts to control behavior—a hypothesis that may open up new pathways for therapeutic and preventative strategies.

The use of addictive drugs is a growing global public health problem. Drug addiction develops with persistent drug use, and individuals who are vulnerable to developing addiction may do so as quickly as within their first year of use (1). Improving our understanding of the brain systems that are implicated in resilience to developing addiction, or “substance use disorder” according to the *Diagnostic and Statistical Manual DSM-5* (2), would offer new opportunities for its prevention and treatment, particularly in young people at risk for addiction.

Over the past decade, a large body of work has elucidated how addictive drugs affect brain function, specifically that of different striatal subsystems that have been critically implicated in the development of addictive behavior (3). Importantly, not everyone who uses drugs, even highly addictive drugs such as amphetamines or cocaine, makes the transition to addiction (1). A family history of drug addiction has been shown to confer an eightfold increased risk of developing addiction (4), and this likely relates to a combination of genetic (5) and environmental (6) risk factors. However, relatively little is known about factors that protect people from developing addiction: i.e., confer resilience. The term “resilience” refers to protective factors that help individuals to successfully cope with or overcome exposure to significant risk, adversity, or potentially harmful environments (7). These factors may include personality traits and attitudes, supportive environments (8), and neural systems that are more robust to, or able to compensate for, adverse exposures (9). Much of the neuroscientific research on resilience has been examined in adolescence (10), which is a period of heightened vulnerability and neural plasticity (11). While studies of adolescents are important to inform the development and implementation of preventative strategies, we also need to understand how resilience operates in vulnerable adults as the compensatory mechanisms that have been characterized in adolescents do not necessarily translate into adulthood. For example, the cognitive deficits frequently measured in adolescents with a family history of addiction (12, 13) are not seen in adults with familial risk (14, 15), which may suggest that adults manage to counteract their vulnerabilities more effectively than adolescents by recruiting compensatory brain systems (16–18). Understanding how potentially vulnerable adults defy the risk of addiction may inform the development of more effective treatment strategies for individuals affected by the disorder.

A more compelling illustration of resilience to drug addiction is demonstrated by individuals who regularly use highly addictive drugs like cocaine but do not succumb to addiction and maintain successful lives (19). These individuals have been described as highly intelligent (20), well-educated, affluent, and socially well-integrated (21). They use drugs in a manner that avoids conflict with their private and professional commitments (21). These recreational users are less visible and thus less well-studied than their addicted counterparts, who already have a diagnosis of substance use disorder and are generally recruited through referrals from treatment services. The available evidence, however, indicates that the recreational users function at a comparable level to control participants by recruiting compensatory brain systems, which appears to buffer the impact of their drug use (22, 23). It is thus conceivable that this ability to recruit compensatory brain systems represents a resilience factor to developing drug addiction. However, to date, very little is known about the mechanisms by which drugs of abuse interact with addiction vulnerability.

Alteration of dopamine signaling in the basal ganglia is thought to be involved in the development of certain substance-related addictions (24). There is considerable evidence that addiction develops through the progressive engagement of different striatal subsystems along a ventral-to-dorsal gradient (25, 26) via associative learning processes implicated in habit formation. Habits typically arise when goal-directed behaviors become autonomous of the goal through repetition. Drugs of abuse may foster this transition, precipitating the development of compulsive behavior possibly by nonmutually exclusive mechanisms: 1) Chronic exposure to drugs of abuse (contrary to natural rewards) compromises the neural system for goal-directed behavior (orbitofrontal cortex [OFC] and ventromedial prefrontal cortex [vmPFC]), thereby biasing decisions toward drug-related rewards and shifting behavioral control toward the habit system; 2) regular drug use alters the dopamine system and facilitates the ventral-to-dorsal striatal transition from goal-directed to habitual instrumental performance, and 3) chronic drug use weakens cognitive control—the flexible regulation of behavior between goal-directed and habitual actions, which intervenes whenever behavior becomes maladaptive. Drug-induced changes affect interactions between the inferior lateral prefrontal cortex, anterior cingulate cortex, and striatum (27).

We posited that addiction risk and resilience would be associated with critical changes in the functioning of these corticostriatal systems, and we therefore investigated resting-state corticostriatal connectivity in individuals with familial risk for addiction, or those engaging in regular drug use, and the interaction of these factors. As addiction develops in combination of drug use and predisposing factors, we hypothesized that familial characteristics (as defined by risk factors shared by family members) in interaction with drug-related effects are critical for the transition to addiction in at-risk individuals. We deliberately focused on stimulant drugs because of their direct effect on the midbrain dopamine system that modulates corticostriatal function, resulting in their high addiction liability. We collected resting-state brain activity in individuals with familial risk of addiction, of whom half were addicted to stimulant drugs, and the other half were their unaffected biological siblings. We compared these participants with individuals with no family history of addiction, of whom half had a personal history of regular stimulant drug use (but not a formal diagnosis of addiction), while the other half did not. Our specific hypotheses were that an increased vulnerability for addiction would be reflected in functional dysconnectivity in frontostriatal pathways implicated in goal-directed behavior and prefrontal control (28) and that resilience to developing addiction would be associated with compensatory responses within the goal-directed system or dorsal-striatal subsystems implicated in habit regulation (29).

## Results

**Fig. 1.**
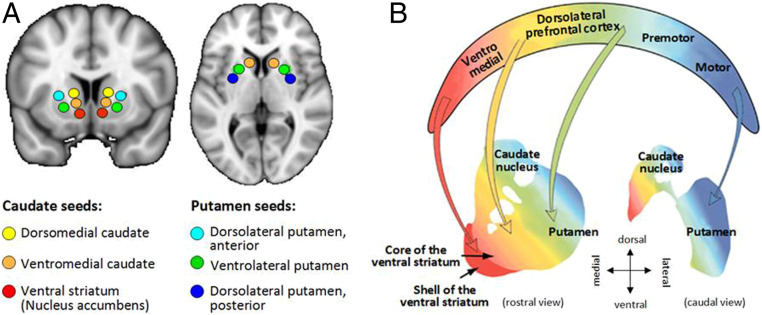
(*A*) Illustration of the six striatal regions of interest in the analysis. These seed regions were first described by DiMartino et al. (30) using anatomical labels, but we decided to use functionally relevant terms to make them comparable with the contemporary literature. Ventral striatum (Nucleus accumbens), ventral striatum inferior; Ventromedial caudate, ventral striatum superior; Dorsomedial caudate, dorsal caudate; Dorsolateral putamen, posterior, dorsal-caudal putamen; Ventrolateral putamen, ventro-rostral putamen; Dorsolateral putamen, anterior, dorsal-rostral putamen. (*B*) Schematic illustration of the key structures within corticostriatal pathways, as previously described (104).

Group characteristics are shown in Table 1. Three of the six striatal seed regions, the ventromedial caudate (vmCAU), ventrolateral putamen (vlPUT), and posterior/dorsolateral putamen (pdlPUT) revealed significant group differences in resting-state functional connectivity (RSFC) within distinct anatomical pathways (Table 2). The topographies of the identified networks are consistent with prior work by DiMartino et al. (30) and the widely described corticostriatal circuitries (31) (Fig. 1).

**Table 1.** Descriptive statistics of study participants, with respect to familial risk and stimulant drug use, if not stated otherwise, shown in means and SD in parentheses

	No familial risk		Familial risk		Familial risk vs. no fam. risk		Drug use vs. no drug use	
	No stimulant use	Stimulant use	No stimulant use	Stimulant use				
Demographics	Mean (±SD)	Mean (±SD)	Mean (±SD)	Mean (±SD)	*F, t,* or χ^2^ *P*		*F, t,* or χ^2^ *P*	
Number	48	25	47	42	162		162	
Gender, % male	63%	48%	51%	95%	3.7	0.056	7.5	0.006
Handedness, % right	85%	84%	89%	81%	0.01	0.934	0.9	0.352
Ethnicity, white: black: Asian: mixed	48: 0: 0: 0	25: 0: 0	36: 4: 2: 5	32: 3: 2: 5	20.7	0.001	0.8	0.560
Age, y	32.4 (±8.8)	28.4 (±6.7)	32.2 (±8.2)	34.7 (±7.7)	2.7	0.103	0.1	0.797
Body mass index	25.2 (±3.3)	24.8 (±4.2)	26.3 (±5.6)	24.7 (±3.9)	0.6	0.451	2.3	0.127
Duration of formal education, y	12.7 (±1.9)	13.4 (±1.8)	12.2 (±2.0)	11.6 (±1.6)	12.2	0.001	0.3	0.586
Verbal intelligence, NART score	112.6 (±7.9)	116.2 (±5.2)	108.6 (±8.9)	110.4 (±7.1)	14.2	<0.001	1.5	0.216
Childhood adversity, CTQ abuse	17.5 (±5.4)	18.5 (±3.9)	24.3 (±10.8)	27.0 (±11.9)	28.5	<0.001	32	0.077
Community Integration, CIQ-II score	17.9 (±3.2)	18.3 (±2.9)	17.6 (±3.4)	14.4 (±3.9)	9.8	0.002	7.1	0.009
Disposable income, £ per month	663.8 (±938.3)	710.4 (±1149.7)	403.8 (±4.13.5)	415.2 (±667.1)	5.0	0.026	0.02	0.883
Alcohol consumption, AUDIT score	3.2 (±2.3)	5.7 (±1.6)	4.0 (±4.6)	11.3 (±10.9)	7.0	0.009	23.7	<0.001
Tobacco, % never/current/past	43/15/42	12/72/16	4/58/38	2/93/5	36.8	<0.001	36.9	<0.001
Cannabis, % never/current/past	79/0/21	0/44/56	26/17/57	0/69/31	30.3	<0.001	70.1	<0.001
Age of onset tobacco use, y	16.2 (±2.8)	16.1 (±4.0)	14.5 (±2.1)	13.1 (±3.0)	19.6	<0.001	2.8	0.094
Age of onset cannabis use, y	17.9 (±4.2)	16.6 (±2.4)	17.8 (±4.3)	15.0 (±2.8)	0.8	0.367	10.7	<0.001
Age of onset stimulant drug use, y	–	19.0 (±2.7)	–	16.4 (±2.5)			4.1	<0.001

The term “stimulant use” does not reflect severity and includes both recreational and addictive use. Verbal intelligence estimated by the National Adult Reading Test (NART), childhood adversity assessed by the abuse score of the Childhood Trauma Questionnaire (CTQ), community integration was evaluated from the Community Integration Questionnaire Version 2 (CIQ-II), and alcohol consumption assessed by the Alcohol Use Disorders Identification Test (AUDIT).

**Table 2.** Regions showing significant functional resting-state connectivity with the striatal seeds depicted in Fig. 1*A*

Seed	Effect	Significant cluster	No. of voxels	Peak MNI coordinates	Brodmann areas
Ventromedial caudate	Familial Risk	OFC, mPFC, rostral ACC,	143	−9, 39, −9	11
		mPFC	21	9, 36, −15	11
		mPFC	19	−6, 66, 6	10
	Stimulant Use	PCC	10	−3, −24, 42	23, 31
	Interaction	IFG, middle frontal gyrus	78	42, 36, 6	45, 46
Ventrolateral putamen	Stimulant Use	Insula	126	−39, 6, −3	48
		Supramarginal gyrus	79	57, −30, 42	40
		Supramarginal gyrus	57	−57, −42, 36	39, 40
	Interaction	ACC	2	9, 30, 18	32, 24
Dorsolateral putamen (posterior)	Interaction	Central opercular cortex	35	57, 3, 9	48, 6
		Superior medial frontal cortex, SMA, cingulate gyrus (middle), insula	10	0, 12, 45	6, 32
			6	15, 9, 39	32, 24
			3	39, 6, 6	13

ACC, anterior cingulate cortex; IFG, inferior frontal gyrus; mPFC, medial prefrontal cortex; OFC, medial and lateral parts of the orbitofrontal cortex; PCC, posterior cingulate cortex; SMA, supplementary motor area.

### Vulnerability-Related Functional Dysconnectivity.

**Fig. 2.**
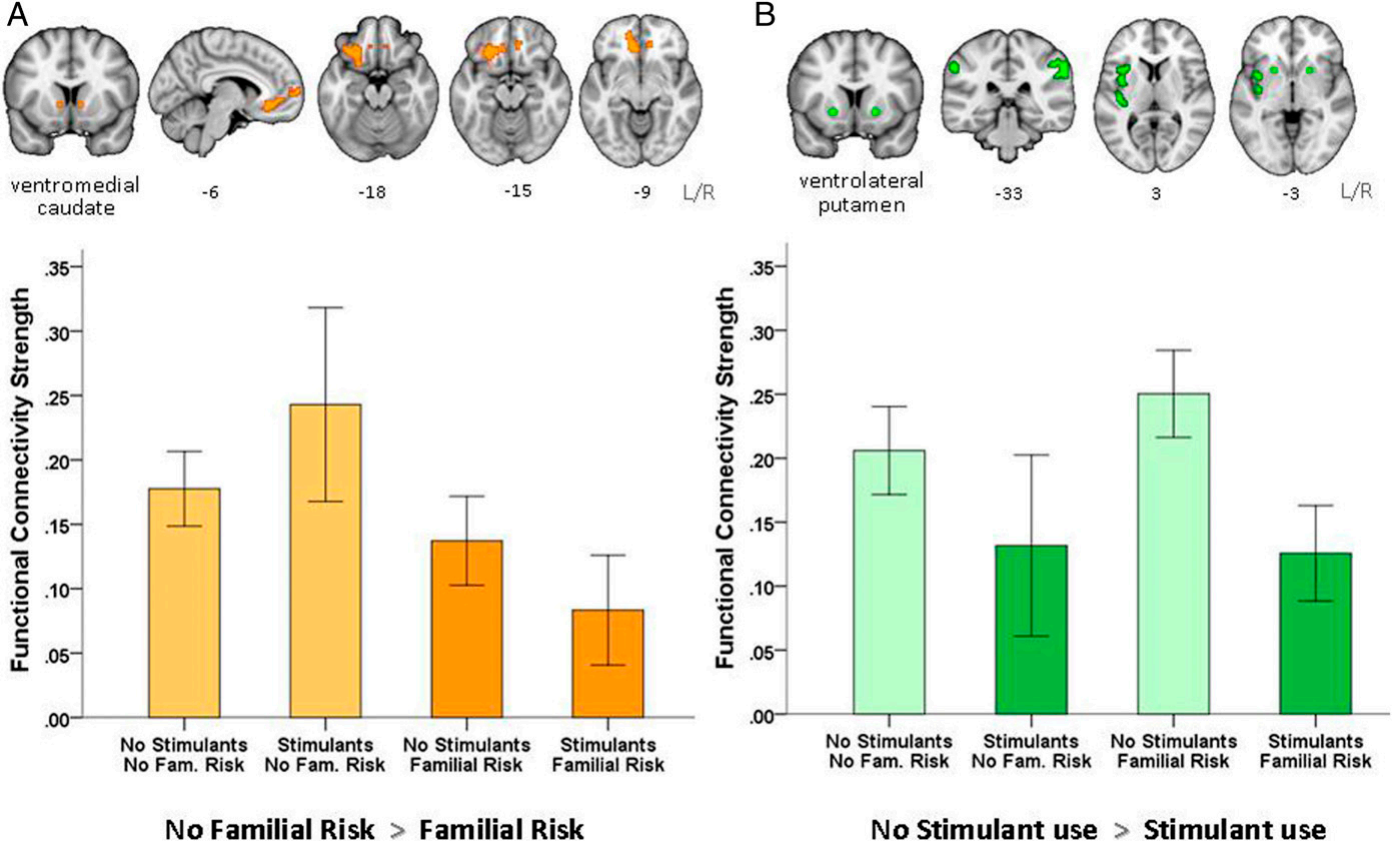
(*A*) Hypoconnectivity between the vmCAU (seed), OFC, mPFC, and rACC is associated with familial vulnerability for developing stimulant drug addiction, as coloured in orange in the brain maps and plotted for each group in the bar chart. Participants with familial risk of addiction showed reduced functional connectivity strength in this network irrespective of stimulant drug use. (*B*) Hypoconnectivity between the ventrolateral putamen (seed), insula, and supramarginal gyrus is associated with regular use of stimulant drugs, as coloured in green in the brain maps and plotted for each group in the bar chart. Participants who use stimulant drugs show significantly reduced connectivity strength in this network irrespective of their familial risk. Error bars denote two SEMs.

Familial risk of addiction was associated with significantly reduced functional connectivity in frontostriatal pathways between the vmCAU seed with the rostral anterior cingulate cortex (rACC), medial, though mainly lateral, OFC, and medial prefrontal cortex (mPFC). These connections survived cluster-level correction (within the whole brain) and (for extracted scores) post hoc statistical correction using a mixed-effect model (β = −0.189, *P* = 0.045). As shown in Fig. 2*A*, the group differences were mainly left-lateralized, and participants with both familial risk and stimulant use (F+S+) showed the greatest reduction in RSFC compared with all of the other groups; participants with familial risk but without stimulant drug use (F+S−) fell midway between F+S+ and F−S− groups. Thus, stimulant use interacts with familial risk further to reduce functional connectivity of cortical (rACC, mPFC, OFC) regions with the vmCAU.

Stimulant use (i.e., whether in recreational users or diagnosed stimulant use disorder) was related to hypoconnectivity in two left-lateralized networks associated with the vmCAU or vlPUT seeds (Fig. 2*B*). Compared with nonusers (S−), stimulant users (S+) showed reduced RSFC between 1) vmCAU and the posterior cingulate gyrus (β = −0.231, *P* = 0.194) and 2) the vlPUT, the insula and the supramarginal gyrus, but only the latter survived post hoc correction (β = −0.299, *P* = 0.045).

The sibling pairs differed significantly from the other two groups in terms of education, income, and age of tobacco use onset (Table 1)—variables that have been considered as preexisting individual and family characteristics which contribute to chronic drug use in adulthood (32, 33). When we added these three variables in our mixed-effect model, the effect of familiality was no longer significant (β = −0.114, *P* = 0.274) while the effect of stimulant use survived (β = −0.366, *P* = 0.020), supporting the notion that socioeconomic factors play a critical part in the addiction vulnerability that family members share.

### Interaction of Familial and Stimulant-Related Risks.

**Fig. 3.**
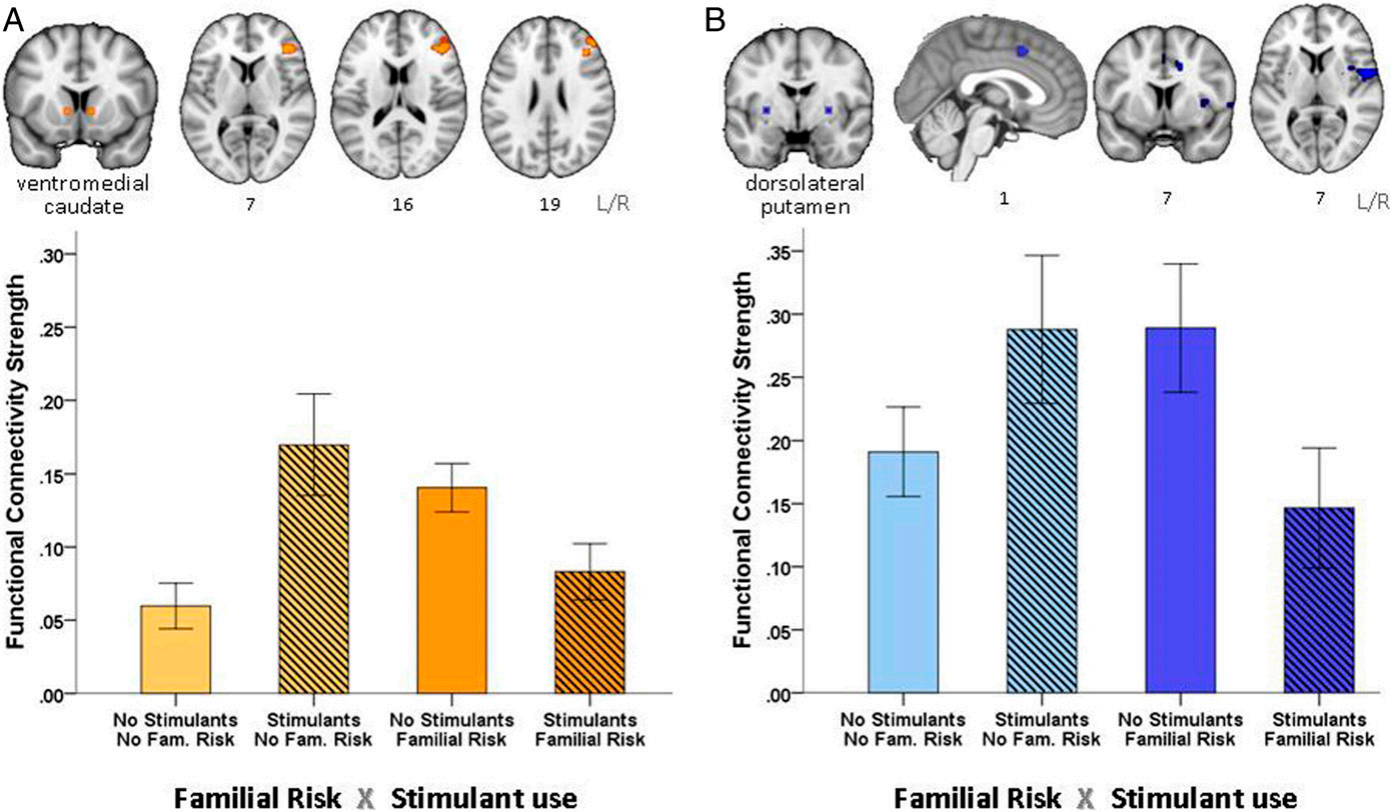
Participants who had an increased risk for addiction, either due to stimulant drug use or a family history of addiction, showed increased functional connectivity in two networks compared to individuals who have such risks and developed addiction, or who do not have these risks and do not use stimulant drugs: (*A*) Increased functional connectivity in the goal-directed, inhibitory control network: vmCAU (seed), inferior frontal and middle frontal gyrus, as coloured in orange in the brain maps and plotted for each group in the bar chart, and (*B*) habitual control network: dorsolateral putamen (seed), central opercular cortex, superior medial frontal, SMA, middle cingulate gyrus, and insula, as coloured in blue in the brain maps and plotted for each group in the bar chart. Error bars denote two SEMs.

Significant interaction effects were observed for three of the six seeds: vmCAU vlPUT, and pdlPUT (Table 2). Individuals who had not developed addiction despite familial risk (F+S−) or regular stimulant use (F−S+), showed hyperconnectivity in key networks compared with those having both (F+S+) or none (F−S−). Both of these groups (F+S−, F−S+) can be described as “resilient.” As shown in Fig. 3, both groups showed significant functional hyperconnectivity between 1) vmCAU and the right inferior frontal gyrus (IFG) and middle frontal gyrus (Brodmann areas [BA] 32 and 6) (β = −0.740, *P* < 0.001), as well as between 2) pdlPUT and several structures, including the central opercular cortex, supplementary motor area (SMA), medial superior frontal cortex, middle cingulate gyrus, and insula cortex (β = −0.482, *P* = 0.020). The significant interaction effects related to the vmCAU and the posterior dlPUT pathways, respectively, were right-lateralized and significantly correlated with one another in the entire sample (*r* = 0.31, *P* < 0.001). However, this correlation was only evident in individuals who did not use stimulant drugs (F−S−: *r* = 0.31, *P* = 0.030; F+S−: *r* = 0.37, *P* = 0.010), and not in stimulant-using participants (F−S+, F+S+). The lack of this relationship in stimulant-using participants (*t* = 1.43, *P* = 0.157) was partly mediated by their pattern of compulsive stimulant drug use, as measured by the obsessive-compulsive drug use scale (OCDUS) (34) (*R*^2^ = 0.38, F_1,65_ = 10.97, *P* = 0.002), suggesting that 1) compulsive stimulant use disrupted the interplay between these two regulatory networks and 2) that the corticostriatal hyperconnectivity in the “resilient” group contributed to their protection from compulsive drug use. The inclusion of education, income, and age of tobacco onset as covariates in the mixed-effect model did not change the significance of the results (vmCAU-IFG β = −0.725, *P* < 0001 and pdlPUT-SMA β = −0.457, *P* = 0.033).

## Discussion

Changes in corticostriatal circuits have been suggested to underlie both the development and the phenotype of drug addiction, possibly driven by distinct mechanisms. Using seed-based correlational analysis in corticostriatal systems in four groups (individuals exposed to stimulant drugs, both with and without clinically diagnosed drug addiction, the siblings of addicted individuals, and healthy control volunteers), we identified distinct, lateralized abnormalities in functional connectivity strength associated with both increased risk of, and resilience to, developing stimulant addiction. The risk of developing addiction (whether due to familial vulnerability or a diagnosis of stimulant use disorder) was associated with significant reduction in functional connectivity (hypoconnectivity) between vmCAU, left OFC, and vmPFC, regions that have been critically implicated in flexible, goal-directed, value-based decision-making (35). Resilience against the risk of developing addiction (either because drug use was not initiated despite increased genetic or social/environmental risk [unaffected siblings] or regular drug use had not transitioned into addiction [recreational stimulant users]) was associated with hyperconnectivity in two regulatory control networks: One network involved the right lateral prefrontal cortex (lPFC) (including the right IFG) and its connections to the vmCAU. This network is implicated in regulating selected actions (36) by down-regulating habitual control over behavior (37). The lPFC should be distinguished from the vmPFC, which is more associated with the representation of goals (28) and exhibits hypoconnectivity in participants with familial risk. The other network involved is the dorsal putamen-SMA, which is critically implicated in sensorimotor function and the transition from goal-directed to habitual control over behavior (38, 39). Resilience thus appears to be associated with hyperconnectivity between different sectors of the prefrontal cortex and the striatum, presumably maintaining regulation over goal-directed behavior and its transition to habitual control.

### Risk of Addiction Is Associated with Hypoconnectivity in vmPFC and Striatal Pathways.

As shown in Fig. 2*A*, familial risk of developing addiction was associated with a significant reduction in functional connectivity within a vmPFC/OFC-striatal network encompassing the vmCAU, mPFC, and adjacent medial and lateral OFC and rACC regions. Hypoconnectivity has generally been associated with functional decline and cognitive impairment, which, in light of the present findings, points toward a weakness in the vmPFC, goal-directed system, possibly biasing behavior toward habitual control. Preclinical studies have shown that lesions and/or pharmacological manipulations of this pathway reduce sensitivity to outcome value and facilitate habit formation (40–42). Indeed, reduced task-related activation in this pathway has repeatedly been reported in drug-addicted adults during maladaptive decision-making (43–45), which may exemplify choices that were not controlled by their consequences. As hypoconnectivity in the goal-directed system is also seen in unaffected siblings, it is possible that this functional weakness may have predated drug-taking, rendering individuals vulnerable to developing drug addiction upon initiation of stimulant drug use. Regular stimulant drug use has been shown to correlate with a reduction of gray matter in the vmPFC (46), suggesting that the likely preexisting underactivity of this system has been exacerbated by chronic stimulant drug use.

The amygdala is also important for modulating both goal-directed and habitual behavior in addiction, based on several animal-based studies (47). However, examining the connectivity of the vmPFC with the amygdala was outside the scope of our seed-based analysis that was focused solely on the striatum.

Importantly, structures implicated in motivational control over behavior, such as the nucleus accumbens, did not reveal abnormalities in functional connectivity. This is consistent with previous research showing normal activation in the ventral striatum during reward anticipation in stimulant-addicted adults (48, 49) and their unaffected siblings (17). Our data thus further confirm the notion that addiction vulnerability is reflected more by dysconnectivity in the network that underlies deliberate action selection rather than the motivational priming of actions.

### Stimulant Use Is Associated with Hypoconnectivity between Ventral Putamen and Insula.

We also found that regular use of stimulant drugs was associated with reduced functional connectivity between the vlPUT, insula (both anterior and posterior parts), and the supramarginal gyrus (Fig. 2*B*), a network associated with emotional awareness (50) and habituation to either pleasant (51) or painful stimuli (52). Hypoconnectivity in this network has previously been reported in cocaine-addicted adults with a high risk for relapse (53). In light of cocaine’s ability to acutely increase activity in this network (54, 55), the reduced functional coupling in regular stimulant drug users might reflect tolerance to the effects of the drug. Reduced functional connectivity between the vlPUT and the insula further supports the notion of compromised interoception in stimulant addiction (56, 57), which may impair intuitive decision-making (58). As bodily feedback has been shown to influence cognitive–affective processes (59) and guide individuals’ choices (60, 61), it could be speculated that reduced functional connectivity in this pathway may in part account for the lack of avoidance responses to aversive events (62, 63) frequently observed in chronic stimulant users and may contribute to the development of compulsive drug-seeking.

### Resilience Against Addiction Is Associated with Hyperconnectivity in Corticostriatal Pathways.

Hyperconnectivity in two regulatory control pathways in resilient individuals may seem paradoxical as these individuals do not meet the criteria for addiction and typically perform within the normal range. However, hyperconnectivity at rest is not thought to be associated with dysfunction but instead to reflect compensatory efforts to meet increased functional demands (64–66). This may explain why unaffected first-degree relatives of addicted individuals show significant overactivation in the lPFC network during tasks of response inhibition (16, 67) and interference control (22, 68) for which hyperconnectivity at rest may be enabling. In this study, addicted individuals (F+S+), whose reduced activation in this network has previously been associated with widespread impairments in a range of executive control tasks (69), show equally strong resting-state connectivity in these networks as their nondrug using peers (F−S−). This may suggest that their regulatory networks are already at maximal capacity compensating for the executive control deficits typically seen in addiction (27). Likewise, the connectivity strength of the putamen-sensorimotor-cortical circuit was not measurably different between addicted individuals and control volunteers. This may seem surprising in light of prior evidence suggesting that stimulant-addicted individuals rely on bilateral recruitment of the putamen-sensorimotor-cortical pathway during simple sensorimotor task performance while, for nondrug using controls, contralateral recruitment suffices (70). Given that the putamen-sensorimotor-cortical network is critically implicated in habit learning, it is tempting to speculate whether bilateral recruitment facilitates the development of pathological habits.

Our data suggest that addiction vulnerability is associated with increased demands on regulatory control systems, to which resilient individuals respond with hyperconnectivity. Compromised control is a hallmark of addiction, and this may not be solely reflected in the connectivity strength of the goal-directed and habit system, but by their interactions. Our data suggest that regular stimulant drug use disrupts the coupling between these two regulatory networks, and the degree of this disruption is partly determined by the severity of compulsive drug use, as measured by the OCDUS scale. Conceivably, hyperconnectivity of these two circuitries in the nonaddicted stimulant drug users might prevent their drug use from developing into a compulsive habit.

A second “top-down” system exerting inhibitory control over goal-directed actions between the dorsal anterior cingulate and the vlPUT (68) was also shown to be hyperconnected in resilient individuals (Table 2), but this effect did not survive post hoc correction. This is noteworthy because the dorsal anterior cingulate has been termed an automatic alarm system (71), in support of habit regulation (72, 73), which would support the notion of resilience being associated with increased strength of regulation over both goal-directed and habitual behaviors.

### Strengths, Weaknesses, and Implications.

Examining functional connectivity within corticostriatal networks across individuals with and without familial risk and stimulant drug use offers a unique opportunity for delineating differences in network function that may underlie vulnerability and resilience to addiction. By including groups in the study that are understudied in addiction, such as individuals with a family history of addiction and recreational drug users, we provide a different perspective on the current addiction literature (74). Most striking is the observation that we did not find differences between the groups in networks associated with the nucleus accumbens, which modulates the reinforcing effects of stimulant drugs. Instead, differences were only seen in networks involved in decision-making (goal-directed and interoceptive decision-making) and behavioral control (anterior cingulate and prefrontal inhibitory control over striatal outflow).

There are, however, several other factors that we have not examined, such as genetic influences, drug use severity, or the concomitant use of other drugs. We therefore want to emphasize that our main aim was not to ascertain the effects of specific drugs on brain connectivity, which has already been the subject of prior work, but to identify the pattern of abnormality associated with drug addiction and addiction vulnerability more generally. We therefore employed a study design in which the regular use of several drugs was restricted to the group that met criteria for addiction and less severe drug use was characteristic of the nonaddicted drug user group. However, all drug-using participants regularly used stimulant drugs, which directly affect the function of dopaminergic striatal pathways.

We acknowledge that a major limitation is that relevant behavioral data are not available to endorse our conclusions made about the functioning of the relevant neural circuitries based on our citation of the extensive functional neuroimaging literature that already exists for both healthy individuals and stimulant drug users. Of course, caution should also be used when interpreting these data because task-induced blood oxygenation level-dependent (BOLD) activation and spontaneous BOLD activation during resting-state functional MRI (fMRI) represent different neuronal processes and are therefore not directly comparable (75).

Although our sample size of 162 was reasonable, the group sizes were unbalanced, and there was relatedness between the familiality (F+) but not the control groups (F−). These limitations are largely due to practical reasons of recruitment, but we have statistically controlled for them and did not include these covariates in the main model to avoid overfitting. We also acknowledge the limitation that participants with familial risk were related whereas participants who were using stimulants were not. We statistically accounted for their relationship by using a mixed-effect model in which we considered clustering of siblings within family as a random effect.

Overall exposure to drugs differs between the addicted and nonaddicted drug users, as would be expected. It also raises the important question whether nonaddicted users may only be at an earlier stage in the addiction cycle or represent an entirely different phenotype. We argue for the latter, suggesting that these nonaddicted drug users show a number of characteristics that render them less vulnerable to addiction, including less compulsive drug use, later onset of drug use (including stimulant drug use), higher socioeconomic status, and the absence of both familial risk and childhood adversity (46). More research is therefore warranted to test the hypothesis that the characteristics seen in this nonaddictive phenotype offer protection against the development of addiction in people who use drugs.

The networks we identified using the striatal seeds are strikingly consistent with previously published research using the same seeds in healthy individuals (30), and, while the absence of a significant role of networks involving the nucleus accumbens was surprising, they otherwise concur with certain contemporary neural models of addiction (26, 76). Our findings are also consistent with previous resting-state studies in addicted individuals reporting reduced functional connectivity in prefrontal networks (77–79), but direct comparisons with prior work are difficult because of differences in the analyses [i.e., the analysis method (80, 81) or location of the seeds (82, 83)] and the factorial design of this study.

## Conclusion

This study of functional resting-state connectivity in 162 participants examined two key contributors to the neural changes associated with familial risk, assessed using a group of sibling pairs of whom one was stimulant-addicted while the other was unaffected, and stimulant drug use, assessed not only in *Diagnostic and Statistical Manual of Mental Disorders, 4th Edition, Text Revision* (DSM-IV-TR)–diagnosed stimulant-addicted individuals (84), but also in control participants regularly using stimulants recreationally. This innovative design not only yielded markers of familial risk and resilience but also revealed their interactions. Familial vulnerability for drug addiction is associated with reduced functional connectivity in networks implicated in instrumental learning and goal-directed decision-making. Stimulant drugs weaken goal-directed control even further via hypoconnectivity in pathways implicated in interoception and negative feedback processing, which may increase the risk of behavior becoming compulsive, even in the face of adverse consequences. Resilient individuals counteract the drive to addiction through hyperconnectivity in two distinct corticostriatal pathways that have been associated with top-down goal-directed inhibitory and automatic–habitual control of behavior, respectively. The present study therefore provides valuable insight into possible interactions between familial risk and stimulant drug use for the regulation of behavioral control and potential disruptions that may occur in the development of addiction, but more research is clearly warranted into the neural basis of the changes in functional connectivity to inform the development of more effective strategies for preventative and therapeutic interventions of addiction.

## Materials and Methods

### Participants and Procedures.

A total of 179 participants were recruited for this study (full details described elsewhere) (46). Briefly, the sample included 50 sibling pairs and 79 healthy control volunteers. The sibling pairs comprised full biological siblings of whom one met the DSM-IV-TR (84) criteria for dependence on cocaine or amphetamines, who have been using stimulant drugs for an average of 16.3 y (±6.7 SD), while the other had no personal history of addiction. Of the 79 healthy control volunteers, none had a family history of addiction, but 27 had been using stimulant drugs regularly for at least 2 y (on average, 8.0 y [±6.1 SD]) whereas the remaining 52 reported no prior experience with drugs (including stimulants). The level of stimulant drug use differed significantly between the two drug-using groups both in terms of the duration of stimulant drug use as well as the compulsive pattern of use. Addicted individuals had used stimulant drugs for a longer duration (*t* = −5.1, *P* < 0.001) and in a highly compulsive manner (*t* = −16.0, *p* < 0.001), as reflected by the OCDUS score (mean 24.4 [±9.2]) whereas the nonaddicted users without a family history of addiction did not (mean 1.2 [±1.7]). Exclusion criteria were a lifetime history of a psychiatric, neurological, or neurodevelopmental disorder, traumatic head injury, and stimulant use for medical reasons.

All participants followed the same protocol, as described elsewhere (46). They consented in writing before undergoing medical review and psychiatric screening, which involved a psychiatric assessment using the Structured Clinical Interview for DSM-IV-TR (85), ascertainment of family history of addiction by a semistructured interview, evaluation of childhood adversity (common in families affected by addiction) (86) using the Childhood Trauma Questionnaire (CTQ) (87), and social integration (which may play a role in resilience) (88, 89) using the Community Integration Questionnaire Version 2 (CIQ-II) (90). Drug and alcohol use was quantified using the Drug Abuse Screening Test (DAST-20) (91) and the Alcohol Use Disorders Identification Test (AUDIT) (92), both of which have demonstrated sensitivity in nonclinical populations. All stimulant drug-using participants completed the OCDUS (34) to assess the degree of how compulsively they used the drug. All addicted individuals (except two) provided a stimulant-positive urine sample; urine samples provided by all other participants were negative. The study protocol was approved by the National Research Ethics Committee (NREC08/H0308/310) and written informed consent was obtained from all participants before study enrollment.

### Acquisition and Preprocessing of Neuroimaging Data.

All participants underwent magnetic resonance brain scans at the Wolfson Brain Imaging Centre, University of Cambridge, United Kingdom, using a Siemens TIM-Trio 3T system with an eight-channel radio frequency head coil array. Participants were instructed to keep their eyes closed, relax, think of nothing particular, and lie still in the scanner during the resting-state fMRI scan. The fMRI data including 261 volumes were acquired with a T2*-weighted echo planar imaging sequence (echo time [TE] = 30 ms, repetition time [TR] = 2,000 ms, flip angle = 78°, within-plane matrix = 64 × 64, field of view [FOV] = 192 × 192 mm, 32 axial-slices with 3-mm thickness and 0.75-mm interslice gap). Whole brain T1-weighted structural images were acquired with a magnetization-prepared rapid acquisition gradient echo sequence (TE = 2.98 ms, TR = 2,300 ms, inversion time [TI] = 900 ms, flip angle = 9°, FOV = 240 × 256 mm, 176 sagittal-slices of 1-mm thickness, isotropic 1-mm^3^ voxels). All scans were screened for abnormal radiological appearance by a specialist in neuroradiology. Neuroimaging data of five participants were unavailable and had to be excluded for 12 owing to poor scanning quality, resulting in a final sample of 162 participants.

### Resting-State fMRI Preprocessing.

The resting-state fMRI data were preprocessed using the FMRIB Software Library (FSL) (version 5.0.9, https://fsl.fmrib.ox.ac.uk/fsl/fslwiki/). The first six volumes were discarded to ensure longitudinal magnetization reaches a steady state, resulting in 255 volumes for each participant. We applied motion correction, removed nonbrain structures and signals related to 10% background noise, performed intensity normalization with the grand mean = 1,000 and spatial smoothing with a 6-mm full width at half maximum Gaussian Kernel (using FSL’s SUSAN). Head motion was evaluated by framewise displacement (FD) (93, 94). Four participants were excluded due to excessive head motion (mean FD >0.5 and over 40% volumes were contaminated with FD >0.5). For the remaining participants, signal-based motion noise removal was further applied to reduce the temporal impact of head motion on resting-state fMRI measures (95). The independent component analysis automatic removal of motion artifacts (ICA-AROMA) is an automated approach combining independent component analysis and machine learning for removing motion artifacts from fMRI signals, which has been shown effective for reducing confounding effects on resting-state functional connectivity and increasing reproducibility of resting-state network identification (96). In this study, we used FSL’s ICA tool (MELODIC) to estimate 100 spatial components for each participant’s fMRI data, and then ICA-AROMA for motion noise classification and removal. Voxel-wise regression was then conducted for nuisance variables, including linear and quadratic trends, average white matter, and cerebrospinal signals of their eroded masks based on individual structural image segmentation by FSL’s FAST. Subsequently, low frequency nonneuronal signals were removed from voxel-wise time series by high-pass filtering (>0.01 Hz) (96). Finally, we denoised and normalized the data from native to standard space using three-step registration. We first aligned the mean functional image to participants’ T1 structural image using boundary-based registration. We then registered the T1 image to the Montreal Neurological Institute (MNI) 152 structural template using linear and nonlinear registration. Finally, we wrapped the whole fMRI data into standard MNI space by combining the above registrations and resampled to isotropic 3-mm voxels with trilinear interpolation.

### Functional Connectivity Analysis and Group Comparisons.

The RSFC estimates were computed using a seed-based approach in this study (97). In light of our prior work, which identified abnormalities in striatal structure associated with stimulant drug addiction (46, 98), we selected six bilateral striatal seed regions that relate to well-defined frontostriatal and corticostriatal networks (30), shown in Fig. 1: ventral striatum (nucleus accumbens: ±9,9,−8), vmCAU (±10,15,0), dorsomedial caudate (dmCAU) (±13,15,9), pdlPUT (±28,1,3), dlPUT (±25,8,6), and vlPUT (±20,12,−3). We generated the seeds with a 3.5-mm-radius spherical regions of interest (MNI coordinates described by ref. 30). The average preprocessed fMRI time series were extracted for each seed and then regressed against other voxels’ time series over the whole brain (using FSL’s FSL_GLM with time series normalized to the unit SD beforehand), which produced a statistical parametric map in terms of Pearson’s correlation—RSFC-r map. The Fisher’s r-to-z transformation was conducted, resulting in an RSFC-z map for each seed and each participant, as the final RSFC outcome measure.

### Second-Level Statistical Analysis.

To test the effects of stimulant use and familiality as well as their interaction on RSFC, RSFC-z maps were entered into the two factorial analysis of covariance (ANCOVA) model as previously described (17) to assess main effects of familiality and stimulant use as well as their interaction on the functional connectivity network of striatal subregions. As stimulant drugs have been shown to affect men and women differentially (99), we controlled for gender as the covariate of no interest. As differential effects have to the best of our knowledge not been found with respect to ethnicity, we did not control for it. The design matrix was generated by FSL’s GLM tool, with four group label columns and one column of demeaned scores for the gender. Nonparametric testing was employed to identify significant clusters related to effects of interest by FSL’s RANDOMIZE with 5,000 permutations and multicomparison-corrected threshold (i.e., threshold-free cluster enhancement corrected *P* < 0.05) (100).

The significant mean cluster RSFC-z values (mean corticostriatal connectivity) identified in the main and interaction effects from the neuroimaging data were imported into Mplus software (101) and analyzed separately using mixed-effect models with the following variables as covariates to control for differences in drug-taking experiences: severity of stimulant use (OCDUS score) (34), duration of stimulant use (years), alcohol consumption (AUDIT score) (92), tobacco use (past/present), and cannabis use (past/present). As the family environment presents a risk factor for addiction (102), we controlled for the shared upbringing of the sibling pairs by assigning siblings pairs the same family identification number and including it as a random effect in the mixed-effect model. This two-step approach to the inclusion of covariates was taken to avoid overfitting in the neuroimaging model.

Demographic and questionnaire data were examined with SPSS (v22, IBM) using a two-way analysis of covariance (ANCOVA) model with the two between-subject factors: “familial risk” (familiality, nonfamiliality) and “stimulant use” (present, absent) (17). Both were included in the same statistical model to concurrently investigate their distinct and interaction effects, while mitigating Type I error. In light of the gender-specific responses to cocaine (103), and that stimulant-using participants are predominantly male, gender was included as a covariate in all analyses. We also applied mediation analysis using the process software tool (v2.13; afhayes.com/index.html) implemented in SPSS to examine the impact of stimulant-related compulsivity (OCDUS) on the regulatory balance between the identified inhibitory and habitual control RSFC networks. The χ^2^ or Fisher’s exact tests were used for the analysis of categorical data. All statistical tests were two-tailed, and a significance level was set at 0.05.
